# Supplementary material for: The extreme yet transient nature of glacial erosion
Source: Nat Commun. 2022 Nov 30;13:7377. doi: 10.1038/s41467-022-35072-0 (PMC9712427; doi:10.1038/s41467-022-35072-0)
Supplement: Supplementary file 1 — Supplementary Information [file 41467_2022_35072_MOESM1_ESM.pdf]

Supplementary Information

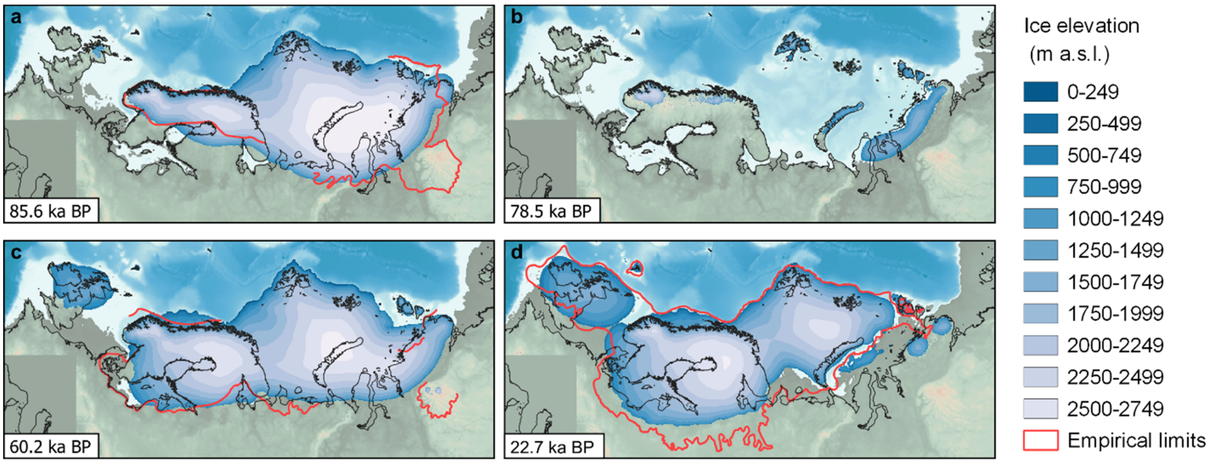

Supplementary Fig. 1 Timeslices of the modelled Weichselian Eurasian ice sheet and contemporaneous sea level compared to chronologically constrained limits for glaciation[1–4].

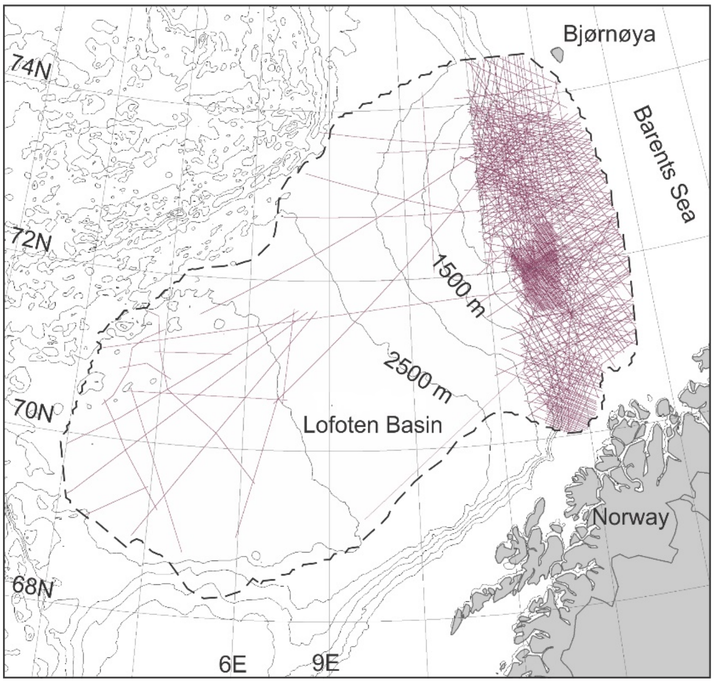

Supplementary Fig. 2 2-D seismic profiles used to constrain the sediment volume within the upper GIII glacial unit of the Bjørnøya trough mouth fan, including a dense network of over 560 industry 2-D seismic profiles covering the outer shelf and upper Bjørnøya TMF, supplemented by 20 seismic profiles that cover the entire TMF. The black dotted polygon represents the external boundary of the deposition area of GIII unit. Contour interval: 500 m.

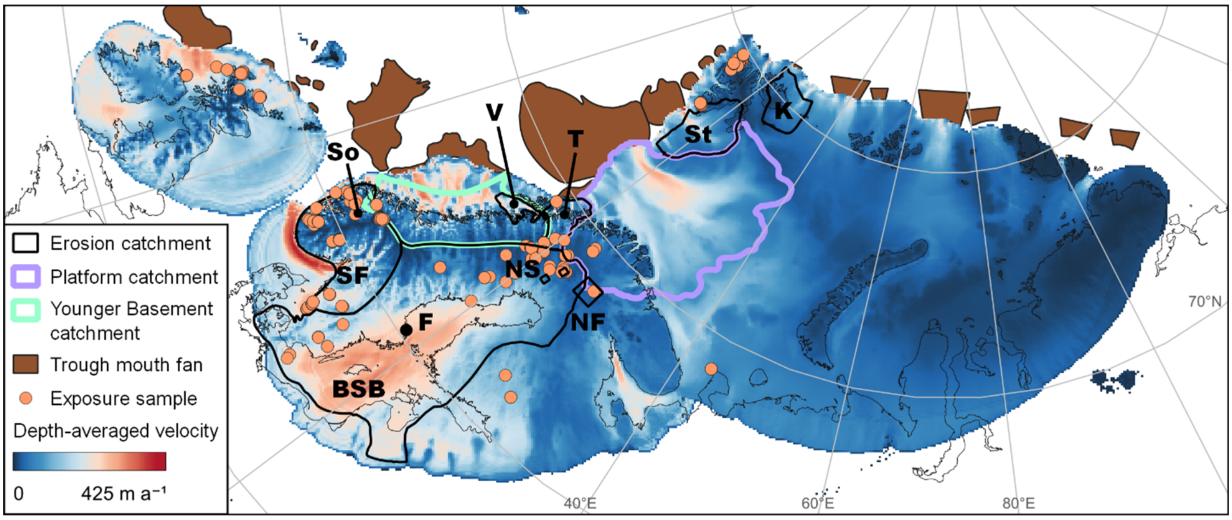

Supplementary Fig. 3 Depth-averaged velocity through the last glacial cycle (123 ka). Back-stripping of trough mouth fan sediments was carried out within erosional catchment areas over Platform and Younger Basement geological provinces. Bedrock cosmogenic exposure samples were used in the scaling of erosion rates against ice discharge in the shield province (expage; Supplementary Data 1). Additional catchments referenced to in this study include: Baltic Sea Basin (BSB), Forsmark (F), Kvitøyrenna (K), Northern Finland (NF), Northern Sweden (NS), Sognefjorden (So), Southern Fennoscandia (SF), Storfjordrenna (St), Troms (T) and Vestfjorden (V).

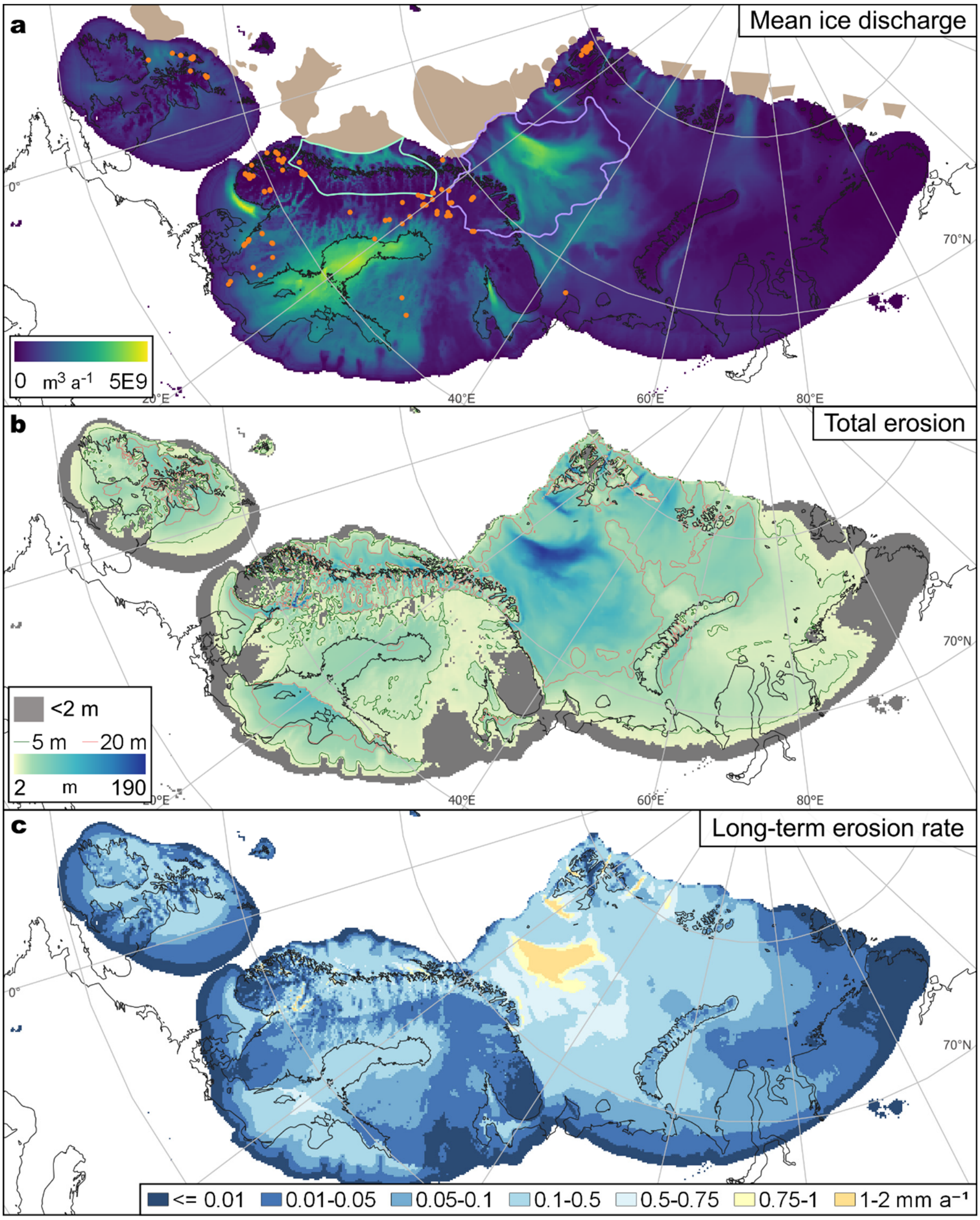

Supplementary Fig. 4 a Mean ice discharge, used to scale patterns of glacial erosion. The stratigraphic sequence and volume of TMF deposits (brown) off the mid Norwegian and western Barents Sea margins were used to determine erosion coefficients for Younger Basement (green) and Platform (purple) provinces, respectively (Fig. 2a). For the Baltic Shield, a coefficient was determined by simulating exposure histories of 249 ^{10}Be bedrock exposure ages (orange dots) to extract a best-fit erosion rate compatible with patterns of modelled ice cover (See Methods). **b** Total glacial erosion during the last glacial cycle (<123 ka). **c** The long-term erosion rate over the last glacial cycle (<123 ka).

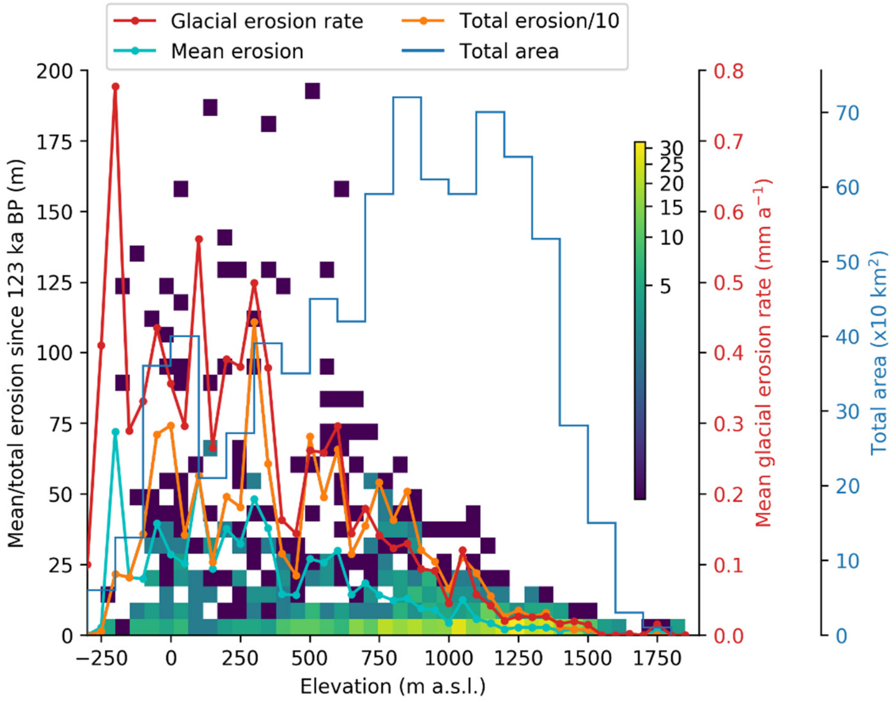

Supplementary Fig. 5 Patterns of glacial erosion with the Sognefjord region, southern Norway (A) The frequency (heatmap), cumulative (orange), mean (turquoise) and rate (red) of modelled glacial erosion by elevation within southern Norway (59.75°N, 62.25°N, 4.75°E, 10°E; Supplementary Fig. 3) since 123 ka BP. The areal extent (blue) within this domain is proportionaly higher between c. 700 – 1400 m a.s.l.

Supplementary Table 1 Modelled estimates for erosion beneath the EISC during the last glacial cycle (<123 ka). Source areas were defined based on the modelled mean pattern of ice discharge (Supplementary Fig. 3). Glacial erosion rates refer specifically to periods when ice covered. In the absence of well constrained and dated ice-sheet history, long-term erosion rates are temporally integrated across glacial and interglacial periods. Empirical long-term rates of erosion were rescaled to derive a scaled glacial erosion rate during the timeframe for which empirical data is available, assuming ice was present over 60% of the catchment for 75% of each glacial cycle and wet-based (i.e. erosive) over 50% of its area[5].

	Modelled							Empirically based	
**Catchment (province)**	**Mean discharge (km^3 a^{-1})**	**Mean duration of ice coverage (a)**	**Eroded bedrock volume (km^3)**	**Source area (km^2)**	**Mean bedrock erosion depth (m)**	**Long-term erosion rate (mm a-1) (<123 ka)**	**Mean glacial erosion rate (mm a^{-1})**	**Long-term erosion rate (mm a^{-1})**	**Scaled glacial erosion rate (mm a^{-1})**
Baltic Sea Basin (Shield/Platform)	1.94	31,330	13,555	879,100	15.42	0.13	0.81	0.02-0.06 (<1 Ma)[6]	0.09-0.26 (<1 Ma)
Southern Fennoscandia (Shield/Younger Basement)	0.722	60,494	2,998	213,000	14.07	0.11	0.24	0.29 (<1.1 Ma) [7]	1.29 (<1.1 Ma)
Mid Norwegian Shelf (Younger Basement)	0.840	59,281	6,311	216,800	29.11	0.24	0.74	0.19 (<2.7 Ma) [5]	0.84 (<2.7 Ma)
Bjørnøyrenna (Platform)	1.474	44,605	35,087	594,000	59.07	0.48	1.48	0.2 - 0.3 (<0.7 Ma) [8]	0.89 - 1.33 (<0.7 Ma)
Kvitøyrenna (Platform)	0.599	81,935	1,471	45,700	32.20	0.26	0.44	0.15 – 0.24 (<2.7 Ma) [9]	0.67 – 1.07 (<2.7 Ma)
Troms (Younger Basement)	0.468	70,098	751	35,000	21.46	0.17	0.34	0.02 - 0.05 (<2.7 Ma) [10]	0.08 - 0.22 (<2.7 Ma)
Storfjordrenna (Platform)	0.785	70,556	3,981	75,900	52.45	0.43	0.80	0.4 (<2.3 Ma) [11]	1.78 (<2.3 Ma)
Total EISC (mixed)	0.873	32,083	126,518	7,186,700	17.60	0.14	0.65	-	-

Supplementary Table 2 Empirical volumetric derivations of long-term erosion rates beneath the EIS, with timeframes adjusted to account for ice-covered periods only. Catchment locations are marked in Supplementary Fig. 3.

Province	Area	Mean ice discharge (modelled) ($x10^9$ m^3 a^{-1})	Source area bedrock volume (km^3)	Glacial erosion rate (mm a^{-1})	Timeframe	Source
Younger Basement	Sognefjorden	0.42569	5,400	0.4	1 Ma during last 2.57 Ma	Nesje & Sulebak [12]
	Southern Fennoscandia	0.72216	35,295	0.67	<1.1 Ma	Hjelstuen et al. [7]
	West Norwegian Shelf (Naust Fm)	0.84017	83,700	0.84*	<2.7 Ma	Dowdeswell et al. [5]
	Vestfjorden	1.59846	330	1.7	35-11.5 ka BP	Laberg et al. [13]
	Troms	0.46849	2,000	0.08 - 0.2*	<2.7 Ma	Rydningen et al. [10]
Platform	Storfjordrenna	0.78481	8,000	0.89*	0.2 Ma (GIII)	Hjelstuen et al. [11]
	Bjørnøyrenna	1.47432	86,700 - 106,000	0.9 - 1.3*	<0.7 Ma (GIII)	Laberg et al. [8]
	Bjørnøyrenna	1.47432	62,105	2.36*	<0.2 Ma (GIII)	This study
	Kvitøyrenna	0.59877	560 - 630	0.06 – 0.11*	<0.7 Ma (GIII)	Lasabuda et al. [9]
	**Area**	**Mean ice discharge (modelled) ($x10^9$ m^3 a^{-1})**	**Total erosion (m)**	**Glacial erosion rate (mm a^{-1})**	**Timeframe**	**Source**
Baltic Shield	Northern Sweden (Area 1)	0.45761	35 (valleys)	0.18	0.2 Ma during last 2.75 Ma†	Hall et al. [14]
	Northern Sweden (Area 3)	1.18311	50 (valleys)	0.25	0.2 Ma during last 2.75 Ma†	Hall et al. [14]
	Northern Finland	0.34341	20	0.1	0.2 Ma during last 2.75 Ma†	Hall et al. [15]
	Forsmark (SE Sweden)	3.42707	1.6-3.5 (simulated)	0.06-0.14 **	100 ka	Hall et al. [16]

* Assuming ice is present over 60% of the catchment for 75% of each glacial cycle duration and wet-based over 50% of its area[5].

** Using our modelled duration of 25.7 ka of ice coverage during the last glacial cycle.

Supplementary Table 3. Principal parameters, constants and values (or ranges) used in the ice-sheet model.

**Parameter**		**Value**	**Units**
g	Gravity	9.81	m s^{-2}
ρ	Density of ice	910	kg m^{-3}
ρ_w	Density of sea water	1028	kg m^{-3}
N	Glen flow-law exponent	3	
A_{weert}	Weertman sliding parameter	7.5×10^{-12}	
γ	Sub-melt sliding coefficient	1	K
m	Sliding-law exponent	1 – 3	
SF	Sliding factor	5	
A_s	Sliding-law coefficient	1.8×10^{-5}	m kPa^{-3} a^{-1}
A_0	Deformation enhancement	75	
a	Material constant		
	T* < 263.15	1.14×10^{-5}	Pa^{-3} a^{-1}
	T* ≥ 263.15	5.47×10^{10}	Pa^{-3} a^{-1}
Q	Creep activation energy		
	T* < 263.15	60×10^{3}	J mol^{-1}
	T* ≥ 263.15	139×10^{3}	J mol^{-1}
A_c	Calving parameter	1.07-29.4	a^{-1}
DDF	Degree day factor	0.005	mm $°C^{-1}$ d^{-1}
T	Temperature	-	K
T^*	(pressure melt corrected)	$T – 8.7 \times 10^{-4}H$	K
a_{max}	Albedo coefficient	8.2×10^{-10}	m^3 W^{-1} s^{-1}
T_{max}	Maximum albedo-temperature	4	°C
$T_{min,sum}$	Minimum albedo-temperature	-14	°C
p	T_{min} exponent	1.3	
$T_{snow-rain}$	Snow-rain threshold	1.0	°C
R	Universal gas constant	8.314	J mol^{-1} K^{-1}
k_i	Thermal conductivity	2115.3 + 7.93 (T-273.15)	J m^{-1} K^{-1} a^{-1}
C_p	Specific heat capacity	3.1×10^{8} exp(-0.0057T)	J kg^{-1} K^{-1} a^{-1}
G	Geothermal heat flux	15 – 705	mW m^{-2}
D	Flexural rigidity	5.0×10^{20}	N m
δx_i	Finite difference interval	1×10^{4}	m
	Central meridian	73	°E
	Latitude of origin	90	°N
x_{min}	Domain dimensions	-4750000	m<br>Lambert azimuthal equal area (central meridian 73°E)
x_{max}		1600000	
y_{min}		-3000000	
y_{max}		-190000	

Supplementary References

1. Svendsen, J. I. *et al.* Late Quaternary ice sheet history of northern Eurasia. *Quat. Sci. Rev.* **23**, 1229–1271 (2004).
2. Patton, H. *et al.* Geophysical constraints on the dynamics and retreat of the Barents Sea Ice Sheet as a palaeo-benchmark for models of marine ice-sheet deglaciation. *Rev. Geophys.* **53**, 1051–1098 (2015).
3. Hughes, A. L. C., Gyllencreutz, R., Lohne, Ø. S., Mangerud, J. & Svendsen, J. I. The last Eurasian ice sheets – a chronological database and time-slice reconstruction, DATED-1. *Boreas* **45**, 1–45 (2016).
4. Stroeven, A. P. *et al.* Deglaciation of Fennoscandia. *Quat. Sci. Rev.* **147**, 91–121 (2016).
5. Dowdeswell, J. A., Ottesen, D. & Rise, L. Rates of sediment delivery from the Fennoscandian Ice Sheet through an ice age. *Geology* **38**, 3–6 (2010).
6. Hall, A. & van Boeckel, M. Origin of the Baltic Sea basin by Pleistocene glacial erosion. *GFF* **142**, 237–252 (2020).
7. Hjelstuen, B. O., Nygård, A., Sejrup, H. P. & Haflidason, H. Quaternary denudation of southern Fennoscandia – evidence from the marine realm. *Boreas* **41**, 379–390 (2012).
8. Laberg, J. S., Andreassen, K. & Vorren, T. O. Late Cenozoic erosion of the high-latitude southwestern Barents Sea shelf revisited. *Geol. Soc. Am. Bull.* **124**, 77–88 (2012).
9. Lasabuda, A. *et al.* Late Cenozoic Erosion Estimates for the Northern Barents Sea: Quantifying Glacial Sediment Input to the Arctic Ocean. *Geochem. Geophys. Geosystems* **19**, 4876–4903 (2018).
10. Rydningen, T. A., Laberg, J. S. & Kolstad, V. Late Cenozoic evolution of high-gradient trough mouth fans and canyons on the glaciated continental margin offshore Troms, northern Norway—Paleoclimatic implications and sediment yield. *Geol. Soc. Am. Bull.* **128**, 576–596 (2016).
11. Hjelstuen, B. O., Elverhøi, A. & Faleide, J. I. Cenozoic erosion and sediment yield in the drainage area of the Storfjorden Fan. *Glob. Planet. Change* **12**, 95–117 (1996).

12. Nesje, A. & Sulebak, J. R. Quantification of late Cenozoic erosion and denudation in the Sognefjord drainage basin, western Norway. *Nor. Geogr. Tidsskr. - Nor. J. Geogr.* **48**, 85–92 (1994).

13. Laberg, J. S., Eilertsen, R. S. & Vorren, T. O. The paleo–ice stream in Vestfjorden, north Norway, over the last 35 k.y.: Glacial erosion and sediment yield. *Geol. Soc. Am. Bull.* **121**, 434–447 (2009).

14. Hall, A. M., Ebert, K. & Hättestrand, C. Pre-Glacial Landform Inheritance in a Glaciated Shield Landscape. *Geogr. Ann. Ser. Phys. Geogr.* **95**, 33–49 (2013).

15. Hall, A. M., Sarala, P. & Ebert, K. Late Cenozoic deep weathering patterns on the Fennoscandian shield in northern Finland: A window on ice sheet bed conditions at the onset of Northern Hemisphere glaciation. *Geomorphology* **246**, 472–488 (2015).

16. Hall, A. M. *et al. Past and future impact of glacial erosion in Forsmark and Uppland*. 247 (2019).
