# Supplementary material for: The extreme yet transient nature of glacial erosion
Source: Nat Commun. 2022 Nov 30;13:7377. doi: 10.1038/s41467-022-35072-0 (PMC9712427; doi:10.1038/s41467-022-35072-0)
Supplement: Supplementary file 3 — Description of Additional Supplementary Files [file 41467_2022_35072_MOESM3_ESM.pdf]

Description of Additional Supplementary Files

File Name: Supplementary Data 1
Description: Bedrock ^{10}Be cosmogenic data analyses used for calibration of the ice sheet / glacial erosion model

File Name: Supplementary Data 2
Description: Matlab code used for analyses of the cosmogenic exposure dataset (Supplementary Data 1).
